# Evolution of defense and herbivory in introduced plants—Testing enemy release using a known source population, herbivore trials, and time since introduction

**DOI:** 10.1002/ece3.6288

**Published:** 2020-05-05

**Authors:** Claire R. Brandenburger, Martin Kim, Eve Slavich, Floret L. Meredith, Juha‐Pekka Salminen, William B. Sherwin, Angela T. Moles

**Affiliations:** ^1^ Evolution and Ecology Research Centre School of Biological, Earth and Environmental Sciences University of New South Wales Sydney NSW Australia; ^2^ Stats Central Mark Wainwright Analytical Centre University of New South Wales Sydney NSW Australia; ^3^ Natural Chemistry Research Group Department of Chemistry University of Turku Turku Finland

**Keywords:** enemy release, herbivore preference, herbivory, introduced plants, leaf traits, plant defense, plant–herbivore interactions, rapid evolution, source population, time since introduction

## Abstract

The enemy release hypothesis is often cited as a potential explanation for the success of introduced plants; yet, empirical evidence for enemy release is mixed. We aimed to quantify changes in herbivory and defense in introduced plants while controlling for three factors that might have confounded past studies: using a wide native range for comparison with the introduced range, measuring defense traits without determining whether they affect herbivore preferences, and not considering the effect of time since introduction. The first hypothesis we tested was that introduced plants will have evolved lower levels of plant defense compared to their source population. We grew South African (source) and Australian (introduced) beach daisies (*Arctotheca populifolia)* in a common‐environment glasshouse experiment and measured seven defense traits. Introduced plants had more ash, alkaloids, and leaf hairs than source plants, but were also less tough, with a lower C:N ratio and less phenolics. Overall, we found no difference in defense between source and introduced plants. To determine whether the feeding habits of herbivores align with changes in defense traits, we conducted preference feeding trials using five different herbivore species. Herbivores showed no overall preference for leaves from either group. The second hypothesis we tested was that herbivory on introduced plant species will increase through time after introduction to a new range. We recorded leaf damage on herbarium specimens of seven species introduced to eastern Australia and three native control species. We found no change in the overall level of herbivory experienced by introduced plants since arriving in Australia.

**Conclusion:**

In the field of invasion ecology, we need to rethink the paradigm that species introduced to a new range undergo simple decreases in defenses against herbivores. Instead, plants are likely to employ a range of defense traits that evolve in both coordinated and opposing ways in response to a plethora of different biotic and abiotic selective pressures.

## Graphical abstract

We aimed to quantify changes in herbivory and defense in introduced plants while controlling for some factors that might have confounded past studies. We compared the introduced Australian beach daisy to its South African source population to measure seven defence traits (corroborated with herbivore preference feeding trials), and we used herbarium specimens of another seven introduced species to track herbivory over time. We found many changes in defense traits but no overall difference in defense, and no changes in herbivory over time. We show that species introduced to a new range do not always undergo a simple decrease in defense against herbivores.

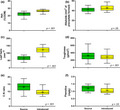

## 1 INTRODUCTION

One of the most prominent explanations for the success of introduced plants is the enemy release hypothesis (Keane & Crawley, 2002), in which introduced species’ escape from natural enemies is predicted to allow reduced allocation to defense, and increased allocation to growth and reproduction (Blossey & Notzold, 1995). These ideas have been exceedingly influential in the field of invasion ecology, with over 950 studies published since the early 1990s (Web of Science, 19 February 2020). Even though many studies have tested the predictions of the enemy release hypothesis (e.g., Chun, van Kleunen, & Dawson, 2010;Colautti, Ricciardi, Grigorovich, & MacIsaac, 2004;Liu & Stiling, 2006;Wolfe, 2002), only about half of these studies’ findings are consistent with the idea that introduced plants escape from their natural enemies (Colautti et al., 2004;Keane & Crawley, 2002). Our overall aim was to consider some novel factors which may be underpinning these idiosyncratic outcomes. The approach we used was testing for evolutionary changes in defense and herbivory in introduced species while controlling for three possible confounding elements of previous studies.

### 1.1 Using a wide native range confounds comparisons of native versus introduced plants

**FIGURE 1:**
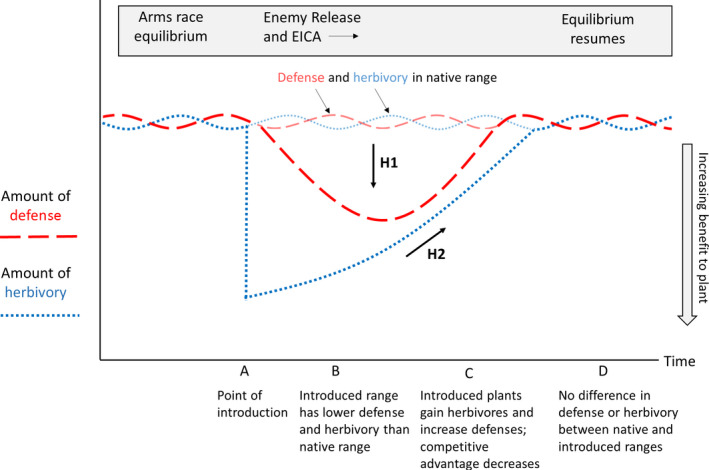
A diagrammatical representation of the changes in defense and herbivory theorized to occur when a plant species is introduced to a new range. In their native range (before point A), plant species are thought to experience an Arms Race Equilibrium of fluctuating herbivory (dotted blue lines) and defense (dashed red lines) as they interact with herbivores (Ehrlich & Raven, 1964). After introduction to a new range (A), introduced species are predicted to experience a significant decrease in herbivory, and a subsequent reduced allocation to defense (enemy release) which could then allow increased allocation to growth and reproduction and thus higher competitive success (B). Over time, however, herbivores in the new range could adapt to the introduced plant, resulting in increasing herbivory and an increasing allocation to defense (C). This may ultimately result in resumption of the Arms Race Equilibrium (D). Within this framework, we predicted that plants would have lower defenses (H1) and increasing herbivory (H2) after introduction to a new range

The first possibility we investigated is that differences between source and introduced populations could be obscured because studies use too wide a native range in their comparisons of native and introduced plants (Dlugosch & Parker, 2008;Liao, Zheng, Lei, & Feng, 2014). A review of common‐environment studies (Colautti, Maron, & Barrett, 2009) shows that comparisons between native and introduced plants can be substantially confounded by among‐population variation in either range. For example, Colautti et al. (2009) showed that without including the effect of latitudinal variation in analyses, introduced plants are found to be larger than native ones, but that including the effect of latitudinal variation rendered this difference nonsignificant. This is because sampling across a broad range of a species’ distribution can introduce a large amount of variation to an analysis. This variation could obscure comparisons between introduced plants in their new and home ranges, which in turn could lead to mixed outcomes in tests of enemy release. To address this problem, microsatellite data (Rollins et al., 2013) were used to pinpoint the actual source population for *Arctotheca populifolia* for use in our common‐environment experiments (full details in Brandenburger, Sherwin, et al., 2019). This novel approach allowed us to provide the first test of evolutionary changes in defense traits of an introduced plant species in comparison with a single known source population. In line with the enemy release hypothesis, we predicted that introduced plants will have evolved lower levels of plant defense compared to the source population (Figure 1).

### 1.2 Herbivore preference trials are necessary to corroborate defense trait measurements

A second possible explanation for idiosyncratic outcomes in tests of the enemy release hypothesis is that the defense traits measured might not be the traits that affect herbivore preferences. Plants employ a range of different defense traits, with evidence that multiple trait combinations may actually evolve together (Agrawal, 2011). In addition, different types of defense traits may affect plant susceptibility to different herbivores in different ways (Carmona, Lajeunesse, & Johnson, 2011). Testing enemy release without aligning defense traits and herbivore preference could then lead to spurious results. One way to overcome these confounding effects is to conduct feeding preference trials (Pérez‐Harguindeguy et al., 2003) to determine whether the feeding habits of herbivores align with changes in defense traits: Lower plant defenses should result in higher herbivory and vice versa. To clarify the biological significance of our measured defense traits, we presented several different herbivore species with a choice of a leaf from either source *A. populifolia* or introduced *A. populifolia* and then measured the resulting leaf damage. In the absence of any herbivore data for *A. populifolia* in the source or introduced ranges, we used five common herbivore species from three diverse classes (insecta, gastropoda, and arachnida) to conduct our experiments. Our prediction (in line with our first hypothesis) was that herbivores would prefer the leaves of introduced *A. populifolia* to the leaves of source *A. populifolia.*

### 1.3 Time since introduction affects measurements of herbivory and defense

Finally, we considered the possibility that rapid evolutionary changes occurring in introduced species could mean that tests of enemy release depend on how long after species’ introduction the study was performed. For example, Zangerl and Berenbaum (2005) examined herbarium specimens of wild parsnip *Pastinaca sativa* and found that its chemical defenses declined upon introduction to a new range, but then increased as the plant re‐encountered its specialist herbivore, the parsnip webworm *Depressaria pastinacella*. Studies of enemy release in this species might then have provided different results depending on when they were undertaken. Assessing how introduced species change over time is crucial to understanding their success, and yet surprisingly, a survey of 199 studies on the effects of invading species (Strayer, Eviner, Jeschke, & Pace, 2006) found that 40% of studies did not consider how much time had passed since introduction. A meta‐analysis by Hawkes (2007) attempted to address this by using several species with known occupancy times to compare herbivory in the introduced range to the home range. She found that herbivory in the introduced range increases until about 150 years after introduction, after which it becomes comparable with herbivory in the home range. However, this approach only provides snapshots of different species with a range of different residency times instead of the optimal situation of obtaining a continuous record of herbivory since introduction. Of course, following the course of an introduced species from its initial introduction is not often possible and may be an expensive, time‐consuming, or difficult undertaking. A promising remedy for this situation is the use of retrospective studies using herbarium specimens, an approach currently gaining prevalence in the literature (e.g., Beauvais, Pellerin, Dube, & Lavoie, 2017;Buswell, Moles, & Hartley, 2011;Calinger, Queenborough, & Curtis, 2013;Dalrymple, Buswell, & Moles, 2015;Flores‐Moreno, García‐Treviño, Letten, & Moles, 2014;Lang, Willems, Scheepens, Burbano, & Bossdorf, 2019;Meineke & Davies, 2019). Evidence suggests that despite biases, gaps, and uncertainty that can exist in herbarium data, they can provide crucial information on distribution and population size shifts, physiological and morphological change, and shifts in ecological interactions (Meineke, Davis, & Davies, 2018). Using insect herbivory as a case study, Meineke et al. (2018) found that herbivory on herbarium specimens follows many of the same predictable patterns as found in empirical and theoretical studies of contemporary herbivory. For example, the fact that most herbarium specimens show little or no damage but a few herbarium specimens show heavy damage is a pattern also observed in field data. Therefore, to test how herbivory might change through time since introduction, we used herbarium specimens to assess changes in the amount of leaf area lost for seven plant species introduced to eastern Australia. Our hypothesis was that herbivory on introduced plant species will increase through time after introduction to a new range (Figure 1).

In summary, the hypotheses we addressed were (a) that introduced plants will have evolved lower levels of plant defense compared to the source population and (b) that herbivory on introduced plant species will increase through time after introduction to a new range. We made use of two kinds of study systems to test these hypotheses. To test our first hypothesis, we used a single‐species study because the actual source population has only been identified for one plant species’ introduction to date (Brandenburger, Sherwin, et al., 2019). To test our second hypothesis, we used a multi‐species approach for a broader understanding of how herbivory changes over time.

## 2 MATERIALS AND METHODS

### 2.1 Defense traits and herbivore preference

**FIGURE 2:**
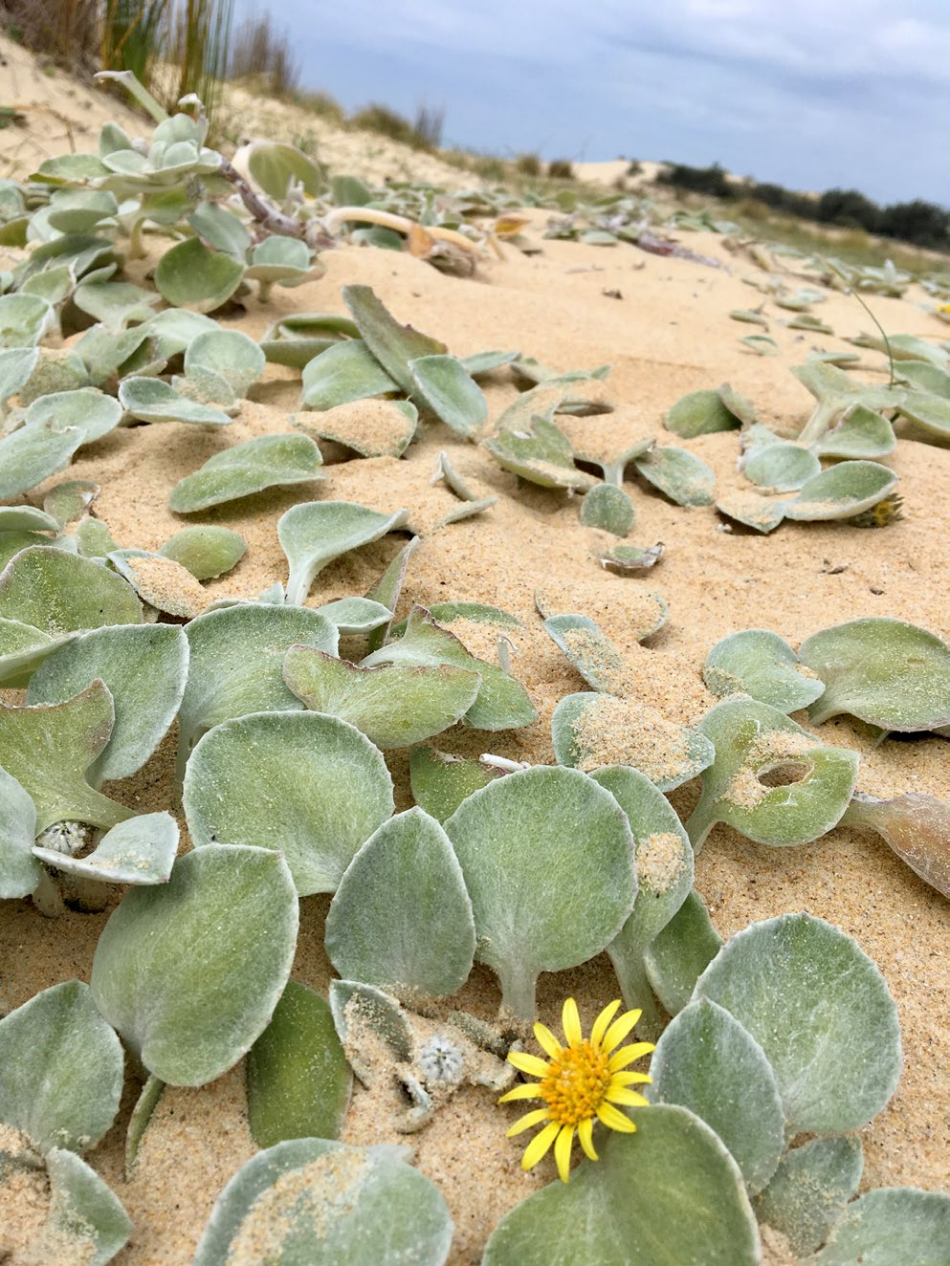
Introduced *Arctotheca populifolia* growing on coastal foredunes on the east coast of Australia

To assess plant defense and palatability traits and associated herbivore preference, we used the South African beach daisy, *Arctotheca populifolia* (P.J. Bergius) Norlindh. This plant is a small, semisucculent, perennial herb from the Asteraceae family (see Figure 2 for photo) and was introduced to Australia in the 1930s (AVH database, 2017;SANBI database, 2017). Following protocols described elsewhere (Brandenburger, Sherwin, et al., 2019), we set up a common‐environment glasshouse experiment using seeds collected from the source population in Arniston, South Africa, and from four populations spanning 600 km of the introduced range along the east coast of Australia. Formal statistical analyses show that Arniston population is at least 10^99^ times more likely to be the source population for the east Australian plants than any of the other population groups sampled throughout South Africa, a result corroborated with STRUCTURE analysis, principal component analysis, differentiation measured by *R_ST,_* and the fact that Arniston is the only population of South African *A. populifolia* that includes all of the rare alleles found in the east Australian *A. populifolia* plants (Brandenburger, Sherwin, et al., 2019;Rollins et al., 2013). To minimize potential confounding due to maternal effects, we used the field‐collected seeds to grow and pollinate a generation of parent plants to produce standardized offspring for our experimental work. We used the standardized seed stock to conduct our experiments over two consecutive growing seasons, the first from December 2013 to November 2014 and the second from November 2014 to October 2015. Each trait was measured on plants from a single growing season. Both growing seasons had equivalent experimental conditions with controlled glasshouse temperatures between 10 and 25°C, daily watering with automatic drippers at 17:00 hr, equivalent natural light exposure, and the same soil composition.

We selected a range of seven physical and chemical leaf traits that are thought to increase plant defense against herbivory or reduce plant palatability for herbivores: leaf toughness, ash, alkaloids, phenolics, cyanogenic glycosides, carbon to nitrogen ratio, and leaf hairs. These traits were chosen because they are often important defenses (Moles et al., 2011). There was no previous information on antiherbivore defenses in *Arctotheca populifolia* and little on other species of *Arctotheca* beyond the suggestion that *Arctotheca calendula* might have cyanogenic properties (Chahl & Kirk, 1975). Thus, in addition to addressing our hypotheses, these data add to our basic ecological knowledge.

Leaf toughness is an important deterrent against herbivory, with tough leaves being less palatable and less digestible for herbivores than soft leaves (Hanley, Lamont, Fairbanks, & Rafferty, 2007). To measure leaf toughness, we used one fresh adult leaf per plant and measured the average pressure (g/mm^2^) required to punch a hole through the leaf using a penetrometer (Chatillon Type 516 push‐pull gauge, 1,000 grams, NY, USA). We used bulldog clips to fasten a leaf between two pieces of Perspex with predrilled holes and then punched three holes per leaf, avoiding the midribs and main veins, to obtain a measure of average lamina toughness.

The ash content of leaves gives us an indication of the amount of insoluble biominerals present, including materials such as calcium oxalates and silicates (Moles et al., 2011). Leaves containing calcium oxalates and silicates are abrasive to herbivores, wear down their mouthparts, and cause digestive problems (Cooke & Leishman, 2011;Franceschi & Nakata, 2005). To measure the ash content of our samples, we combusted 1.0 g of dried leaf material at 550°C for 6 hr and then again for 3 hr (Thermolyne 62700 Furnace), before weighing the remaining material.

Alkaloids are a large group of diverse phytochemicals, including well‐known examples such as caffeine and nicotine. Many alkaloids are repellent or toxic to animals, including both vertebrates and invertebrates (Mithofer & Boland, 2012). Due to the diversity of alkaloids and the ways in which herbivores respond to them, there are many techniques for their analysis (Farnsworth, 1966;Jones & Kinghorn, 2012). High‐pressure liquid chromatography (HPLC) is one method commonly used to analyze alkaloids, but its main purpose is to separate and to quantify specific compounds, for example in tea (Del Rio et al., 2004) and tobacco (Keinänen, Oldham, & Baldwin, 2001). In our study, we required only a comparison of extractable alkaloids in two groups of plants with limited sample sizes (*n* = 22 for source and *n* = 24 for introduced *A. populifolia)*. This can be achieved by extracting plant metabolites, adding a reagent which precipitates the alkaloids, and then quantifying the precipitate (Farnsworth, 1966 p. 245; Sinha & Dogra, 1985; Corsi & Biasci, 1998). We did this by placing 0.5 g of 1mm fresh leaf strips into 10 ml Falcon centrifuge tubes and added hydrochloric acid (HCl, 10 ml 1N solution). We vortexed the tubes until well mixed and then sonicated the samples for 25 min without added heat to break down cell walls. We left the samples to extract overnight. We then centrifuged the tubes at 4,000 RPM and transferred 3 ml of the resulting supernatant into a separate glass vial. We added 0.5 ml of Bouchardat's reagent (also known as Lugol's Iodine or iodine–potassium iodide; 2% I and 4% KI in deionized water [U.S. Pharmacopeia, 2015]), vortexed the vials, and then rested the vials for 3 hr to allow any alkaloids to precipitate. We took photographs of the front and back of each test tube and then used ImageJ software (Rasband, 1997) to measure the average cross‐sectional area of the precipitate formed per test tube (mm^2^).

Phenolics are another important group of structurally diverse compounds used by plants for defense against herbivores, with tannins in particular known to be effective against a wide range of herbivores due to their protein‐binding (Haslam, 1988) and oxidative (Salminen & Karonen, 2011) capacity. We assessed total phenolic content (mg/g dry weight) using a modification of the common Folin–Ciocalteu test at the Natural Chemistry research Group at the University of Turku (Salminen & Karonen, 2011). The amount of phenolics in our samples was too low to test for oxidative activity.

Many plants contain cyanogenic glycosides which release hydrogen cyanide as a deterrent to herbivory (Vetter, 2000). We tested for cyanogenesis using sodium picrate paper which changes color from yellow to red in the presence of cyanogenic glycosides (Brinker & Seigler, 1989). Three plants from each population were randomly selected, and a healthy adult leaf was cut from each. We sliced the leaves into strips and placed them into sterile plastic specimen containers and then suspended a piece of sodium picrate paper inside the lid. We used crushed apple seeds (which are known to contain cyanogenic compounds) as a positive control. After 2 hr, we checked to see whether the color of the sodium picrate paper in each container was still yellow, or whether it had changed to red.

Although nitrogen is a critical nutrient for herbivores, it is usually a very scarce resource, and so leaves with low amounts of nitrogen and/or a high carbon to nitrogen (C:N) ratio are usually less attractive to herbivores than leaves with higher amounts of nitrogen or a lower C:N ratio (Mattson, 1980). We measured leaf carbon and nitrogen content (mg/g) using a 17 LECO TruSpec CN Analyser at the Solid State and Elemental Analysis Unit at UNSW.

Finally, leaf hairs play a significant role in plant defense (Johnson, 1975;Levin, 1973). Leaf hairs can reduce herbivory by leaf chewing or sap‐sucking insects, impede the movement of small herbivores, and may prevent insect oviposition (Hanley et al., 2007). Therefore, the final defense trait we included in our analyses was leaf hair density (leaf hairs/mm^2^). We used clear nail polish to make epidermal impressions of both leaf surfaces from one leaf per plant (*n* = 44). We viewed imprints using an Olympus CX41 microscope at x40 magnification and captured images with an attached digital camera (QImaging MicroPublisher 3.3 RTV). We used the Eyedropper Tool in Adobe Photoshop version 14.0 (Adobe Systems Inc.) to count the number of leaf hairs in each image and then used a stage micrometer to calculate leaf area and convert our leaf hair counts to leaf hair densities.

**FIGURE 3:**
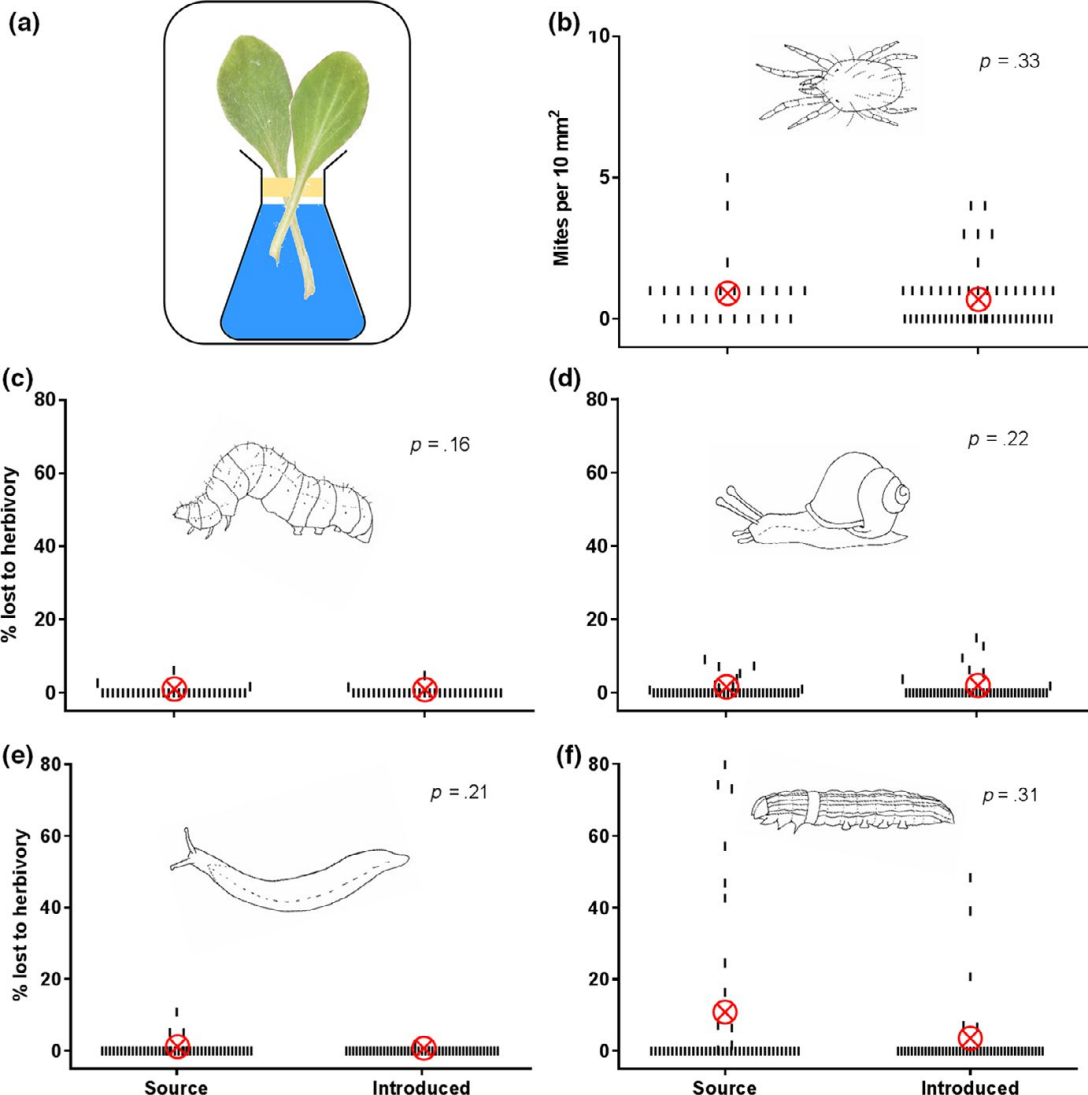
Herbivore preference feeding trials. (a) Representative diagram of the experimental setup for herbivore preference feeding trials (see methods for details). (b) Number of red spider mites (*Tetranychus urticae*) present on source and introduced *A. populifolia* (*n* = 76). Percent of leaf material lost to herbivory in herbivore preference trials containing source and introduced *A. populifolia* for (c) green looper caterpillar (*Chrysodeixis* sp*.*) (*n* = 64), (d) garden snail (*Cornu aspersum*) (*n* = 96), (e) grey field slug (*Deroceras reticulatum*) (*n* = 94), and (f) lily caterpillar (*Spodoptera picta*) (*n* = 96). Each small line represents one measurement; the red circled X symbols represent the average

We assessed herbivore preference for source versus introduced *A. populifolia* by using herbivore preference feeding trials (Pérez‐Harguindeguy et al., 2003). Four common chewing herbivore species were used: green garden looper caterpillar (*Chrysodeixis* sp*.*), garden snail (*Cornu aspersum*), grey field slug (*Deroceras reticulatum*), and lily caterpillar (*Spodoptera picta*). The herbivores were collected from gardens in Sydney, and food was withheld for 48 hr before the feeding trials. The herbivores were almost certainly naïve to *A. populifolia*: Although historic records for the daisy exist, it has not been sighted on any Sydney beach since this project began in 2012. On the day of each feeding trial, we cut fully expanded adult leaves from randomly selected plants from source and introduced *A. populifolia* populations and then scanned them (Canon CanoScan LiDE 200) to quantify preherbivory leaf area using ImageJ software (Rasband, 1997). Feeding trials involved replicates of lidded two‐liter polyethylene terephthalate (PET) bottles, each with a conical flask of water containing one leaf from the source population and one leaf from the introduced population. The flask was centrally placed at the base of the PET bottle, and leaves were separated by a thin strip of masking tape along the flask opening to distinguish the two plant populations (Figure 3a). We placed one individual herbivore on the masking tape to minimize initial preference bias. Trials were held for nine hours according to each species’ feeding periods with snail and slug trials conducted overnight and caterpillar trials conducted from sunrise. We then scanned and analyzed the leaves again to quantify leaf area lost to herbivory. In addition, in the second growing season the glasshouse experienced an outbreak of red spider mite (*Tetranychus urticae*), a sap‐sucking herbivore. This occurred after we had collected our defense data. We capitalized on the opportunity to collect information on this additional herbivore before managing the outbreak. Since the plants were randomly interspersed and mostly touching, we quantified mite presence on leaf undersides as a measure of herbivore preference. This was sampled by using a 10x10 mm quadrat with its corner originating from the center of the leaf. In total, we recorded 426 separate measurements of herbivory across the five species (Figure 3).

### 2.2 Herbivory through time

To assess changes in herbivory through time, we aimed to survey terrestrial vascular plant species that had been introduced to eastern Australia with a known introduction date. Ideally, we would have wanted to include *A. populifolia* in this part of the study but unfortunately, there were not enough herbarium specimens of *A. populifolia* to be able to include it. Instead, we sampled other introduced species from the Australian National Herbarium in Canberra, the National Herbarium of New South Wales in Sydney, and the John T. Waterhouse Herbarium at UNSW Sydney. We excluded agricultural and horticultural introductions, plant cultivars, and species for which biological controls were introduced. We included species for which we could make >60 leaf measurements and which had an introduction date of 1999 or earlier. Seven introduced species satisfied our criteria (Table S1). Three native species from three different families were included to rule out the possibility of any background change in herbivory in the introduced range. We included species with a long (>70 yr) collecting record and with enough specimens to enable us to make comparable leaf measurements with the introduced species (native *n* = 1,005; introduced *n* = 1,170) (Table S1).. We recorded the percentage of leaf area lost on the first five fully expanded, nonsenescent leaves from the roots or base of the plant. All estimates were made by the same observer (MK). Error was minimized by training prior to sampling until observer accuracy was within 2.5% of the output generated by ImageJ software (Rasband, 1997). In total, herbivory was estimated for 2,175 leaves on 435 herbarium specimens.

### 2.3 Data analysis

#### 2.3.1 Leaf traits

Plants came from separate maternal lines or were half‐siblings. For traits with half‐siblings, we used the “lme4” package in R (Bates, Machler, Bolker, & Walker, 2015) to fit models with and without half‐siblings and then tested for any differences using a one‐way ANOVA. We found no effect of half‐siblings (all *p*‐values were >.13) and so we did not include a term for half‐siblings in subsequent models. The introduced Australian populations of *A. populifolia* occur along a latitudinal gradient and so we tested whether there were any significant differences among these four populations using one‐way ANOVAs. We found no significant differences among the Australian populations for any of the leaf traits (Table S2). This is consistent with previous results (23 out of 24 traits) (Brandenburger, Cooke, Sherwin, & Moles, 2019;Brandenburger, Sherwin, et al., 2019). Since there was no significant difference between Australian populations, we compared defense traits between South African (SA) and Australian (AUS) populations with a two‐sample *t* test using the lm() function in the R statistical software, version 3.5.1 (R Core Team, 2019; see Supplementary Data Table S3 for sample size information). For an overall comparison of defense traits, we conducted a random effects meta‐analysis using the “metafor” package in R (Viechtbauer, 2010), using all the traits except C:N (which is useful as an indicator of how palatable the leaf is for herbivores, but is not a defense trait as such). Standardized mean differences using Hedges’ g were combined to create an overall defense difference score.

#### 2.3.2 Herbivore preference

We compared how much leaf material was eaten when each herbivore was presented with a pair of leaves—one from a South African plant and one from an Australian plant (SA vs. AUS)*.* For each of the four chewing herbivores, we fitted a zero‐inflated beta regression model with “proportion eaten” as the response variable and “origin of plant” as the independent variable. Beta regression makes predictions between 0 and 1 and is considered a flexible family for proportion data; zero inflation is required to allow for data located on zero. The zero‐inflated beta regression is a hurdle model, where first the probability of being eaten is modeled and then—conditional on being eaten—the amount of leaf eaten is modeled. Therefore, there are two parameters guiding differences between groups: one for probability of being eaten (SA vs. AUS) and one for amount eaten conditional on nonzero damage (SA vs. AUS). The expected amount eaten is a product of the probability of being eaten and the amount eaten conditional on nonzero damage. The model was implemented using the “gamlss” package in R (Rigby & Stasinopoulos, 2005). Confidence intervals and standard errors of the difference between South African and Australian plants in expected amount eaten were calculated using an ordinary studentized parametric bootstrap procedure. To reconstruct the sampling distribution of the difference in herbivory between South African and Australian plants, the model was re‐estimated 1,000 times on replicate data simulated from the hurdle model (with a term for origin of plant). *p*‐values for a difference between two means were estimated by simulating from hurdle models under the null hypothesis (without a term for origin of plant) and using the difference in expected amount eaten as a test statistic. For mites, we fitted a generalized linear model (GLM) (McCullagh & Nelder, 1989) with a Poisson family response to mite count on each plant and included the origin of the plant (SA vs. AUS) as a predictive factor. A likelihood ratio test determined whether the origin of the plant was a significant predictor of mite count. For an overall comparison of herbivore preference, we conducted a random effects meta‐analysis with all five herbivores using the “metafor” package in R. We used log ratio of means (SA over AUS) as the effect size to combine data that were both proportions (leaf eaten) and counts (mites). For the proportion data (estimated using hurdle models), we estimated the variance of the log ratio of means using a nonparametric bootstrap. For the count data, the variance follows naturally from the distribution of GLM regression coefficients.

#### 2.3.3 Herbivory through time

Many samples showed no damage, and the prevalence of zeros made it impossible to transform the data to an accessible statistical distribution family. Therefore, to assess the effect of herbivory over time, we fitted a hurdle model for herbivory, modeling the probability of being eaten (a binomial family mixed model) and the amount eaten conditional on nonzero damage (a logit‐normal mixed model) (Martin et al., 2005). For the seven introduced species, time was designated as “time since introduction”; for the three native control species, time was designated as “time since first record.” Each species had its own intercept (baseline amount of herbivory). “Species” was included as a fixed effect because there was some correlation between the baseline amount of herbivory and the variable of interest (time): In such circumstances, random effects are known to introduce too much bias (Clark & Linzer, 2015). Species that were introduced in later years were also associated with higher baseline amounts of herbivory, and we wanted to control for that while testing for a trend in herbivory over time since introduction. Both parts of the hurdle model included a random effect to account for specimens on the same sheet, and the model was fitted using the “lme4” package in R (Bates et al., 2015). In both parts of the model, we tested the inclusion of a trend in time for each of the introduced species and for the native species separately, by coding dummy variables. The fitted coefficients for time are log scale ratios of odds in one year compared to odds in the previous year. In the binary model, the odds are the probability of being attacked over the probability of not being attacked, while in the logit‐normal model the “odds” are defined as the proportion eaten over proportion not eaten. Likelihood ratio tests assessed significance of each trend. For the combined effect across both models, the sum of log likelihood ratio statistics was used as a statistic and compared to a chi‐squared distribution with two degrees of freedom. Individual species models followed the same method.

## 3 RESULTS

**FIGURE 4:**
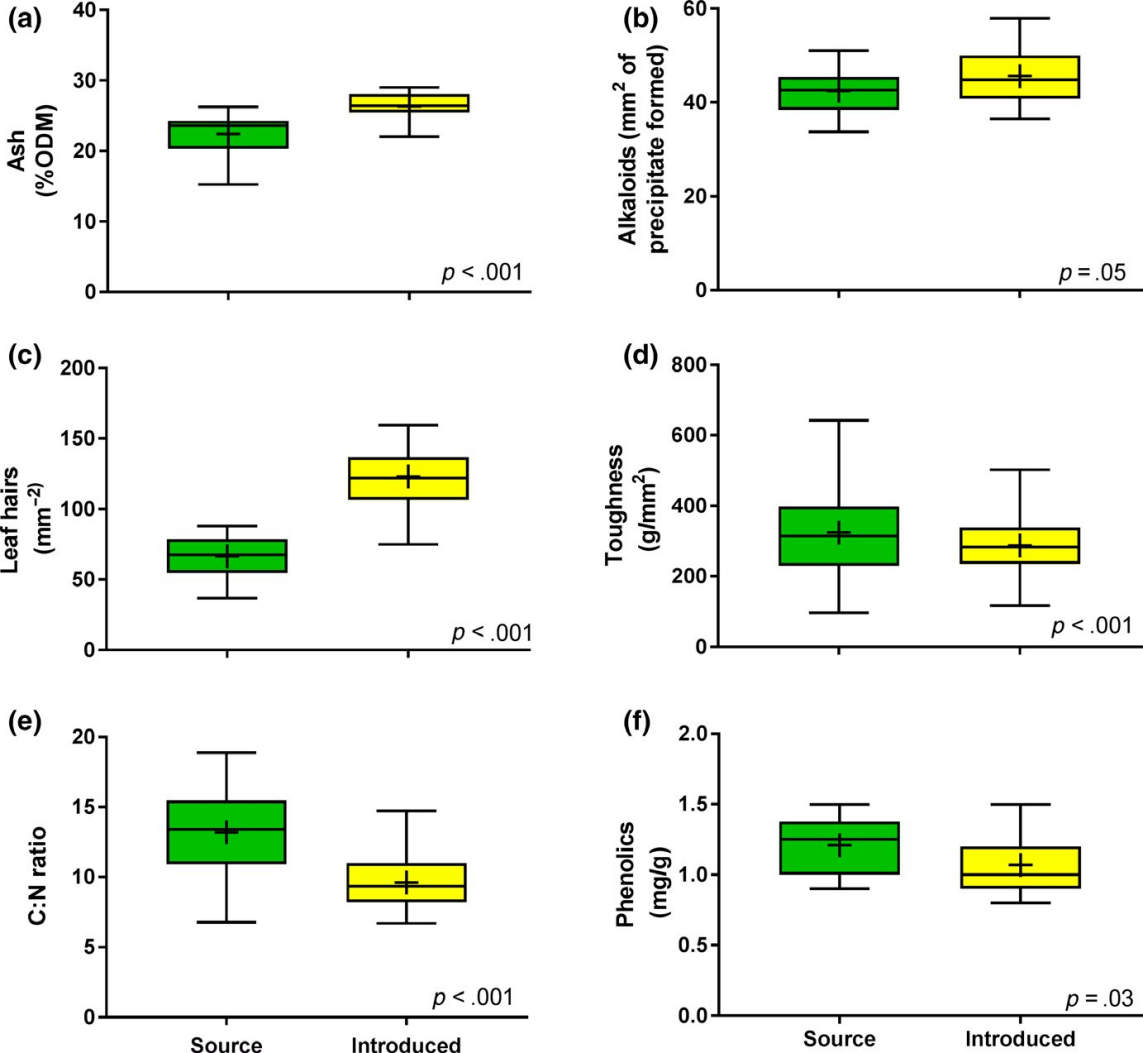
Box plots comparing six leaf traits between South African source plants (green boxes) and Australian introduced plants (yellow boxes). The lower boundary of each box indicates the 25th percentile, a line within each box marks the median, and the upper boundary of each box indicates the 75th percentile. Mean values are indicated with a+ sign. Whiskers above and below each box indicate the minimum and maximum values

Introduced Australian *A. populifolia* plants had rapidly evolved changes in almost every trait that we measured, and yet the results were surprisingly mixed. The leaves of the introduced plants had 17% more ash (*p* < .001), 7% more alkaloids (*p* = .05), and a remarkable 84% more hairs underneath their leaves than the source plants had (*p* < .001) (Figure 4a‐c). However, the leaves of the introduced plants were also 11% less tough (*p* < .001), had a carbon to nitrogen ratio that was 27% lower (*p* < .001), and had less phenolics (*p* = .03) than were the leaves of the source plants (Figure 4d‐f). Notably, levels of phenolics in both groups of plants were very low on a global scale (Kahkonen et al., 1999) and too low to test for oxidative activity; while ash values in both groups of plants were extremely high: In a global study of 301 species from 75 sites (Moles et al., 2011), only three species had higher ash content than we found for *A. populifolia*. No cyanogenic glycosides were detected in either source or introduced *A. populifolia*.

**FIGURE 5:**
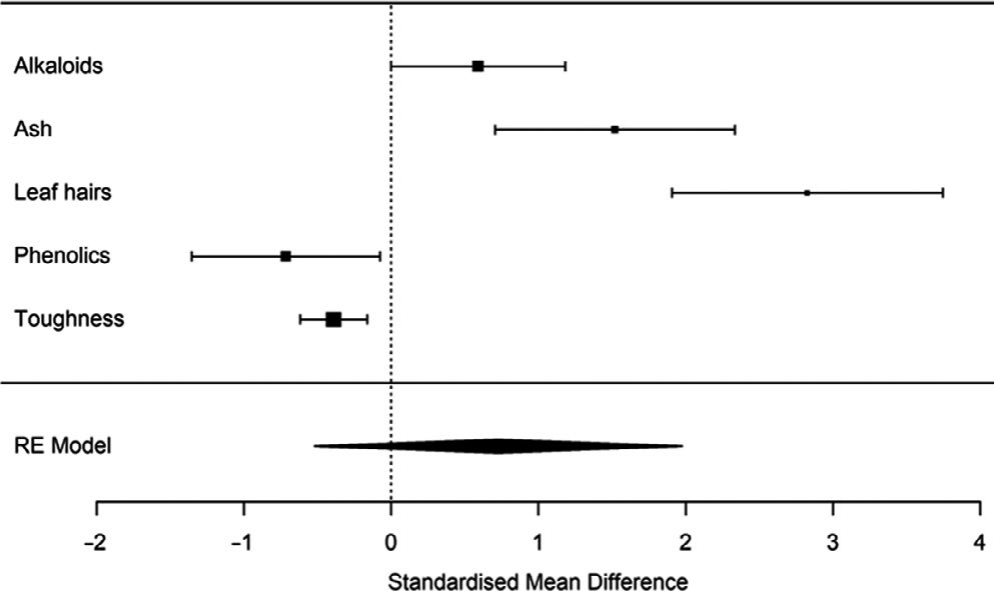
An overall comparison of leaf defense traits using a random effects meta‐analysis. Positive values indicate that Australian plants were better defended than South African plants; negative values indicate that Australian plants were less well‐defended than South African plants. Mean values with 95% confidence intervals are as follows: alkaloids 0.59 [0.00, 1.18], ash 1.52 [0.71, 2.33], leaf hairs 2.83 [1.91, 3.75], phenolics −0.71 [−1.35, −0.08], and toughness −0.39 [−0.62, −0.16]. The score combining each trait and estimated in the meta‐analysis is 0.73 [−0.52, 1.98]

With some traits showing changes consistent with increased defense capabilities in the introduced plants and some traits showing changes consistent with decreased defense capabilities in the introduced plants, the next step was to assess the overall, combined defense capability of introduced *A. populifolia* plants compared to their source population. A meta‐analysis of the five defense traits revealed no significant difference in the overall defense capability of introduced versus source *A. populifolia* (mean effect = 0.73; 95% confidence interval from −0.52 to 1.98; Figure 5). The overall lack of change in defense is not due to a lack of power, as evidenced by the significant changes we found within each individual defense trait. We therefore reject our first hypothesis that introduced plants will have evolved lower plant defense compared to the source population.

**FIGURE 6:**
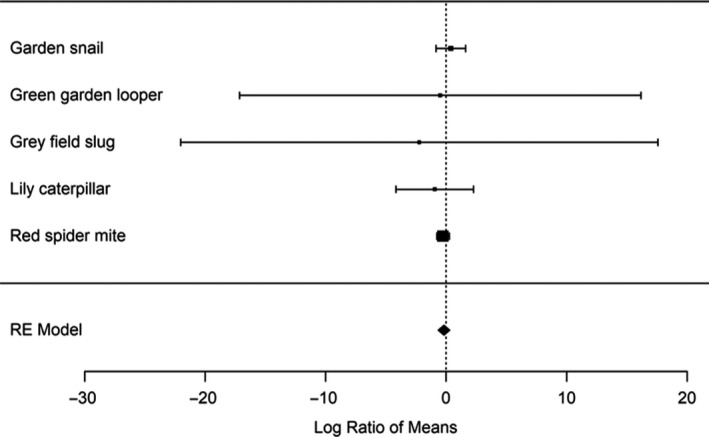
An overall comparison of herbivore feeding preference using a random effects meta‐analysis. Positive values indicate that South African plants had higher levels of leaf damage; negative values indicate that Australian plants had higher levels of leaf damage. Mean values with 95% confidence intervals are as follows: green looper caterpillar −0.49 [−17.14, 16.16], garden snail 0.39 [−0.83, 1.62], grey field slug −2.23 [−22.02, 17.56], lily caterpillar −0.95 [−4.16, 2.27], and red spider mite −0.26 [−0.79, 0.26]. The score combining each trait and estimated in the meta‐analysis is −0.18 [−0.79, 0.29]

Next, we measured how much leaf material the herbivores consumed given the choice of introduced versus source *A. populifolia*. We found no significant difference in the number of red spider mites present on introduced versus source *A. populifolia* plants (*p* = .33; Figure 3b) and none of the four chewing herbivore species showed a preference for consuming either introduced or source *A. populifolia* plants (all p‐values > 0.16; Figure 3c‐f). A meta‐analysis showed that herbivores had no significant preference for leaves from either source or introduced *A. populifolia* (mean effect = −0.19; 95% confidence interval from −0.66 to 0.29; Figure 6). This outcome corroborates the results of our defense trait measurements and shows that *A. populifolia* has not become more susceptible to herbivory in its new Australian range.

**FIGURE 7:**
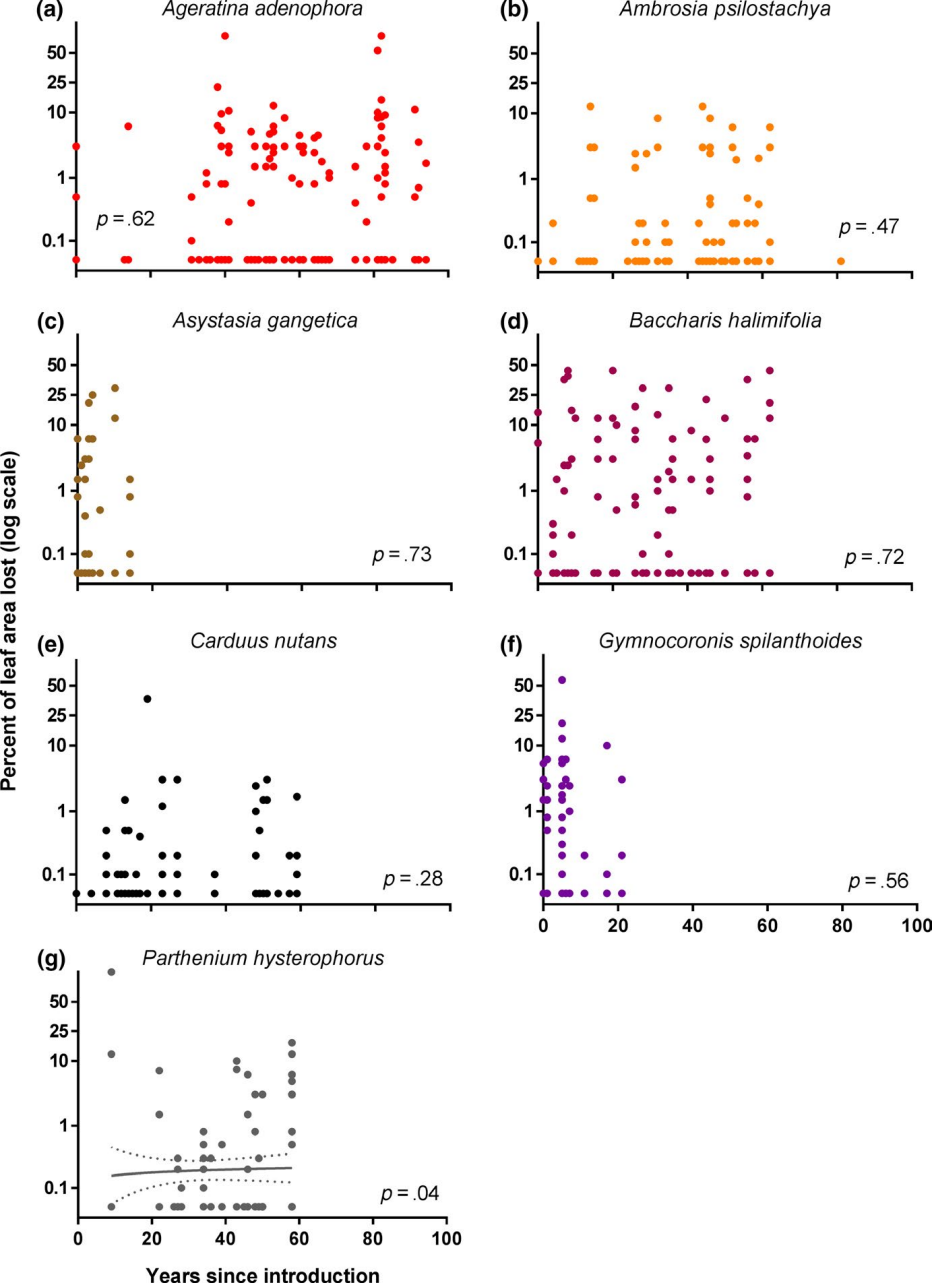
Percent leaf damage (logit transformed) in herbarium specimens of seven individual introduced species in the years following introduction to a new range. For *Parthenium hysterophorus,* a semilog line of best fit with 95% confidence bands is shown

**FIGURE 8:**
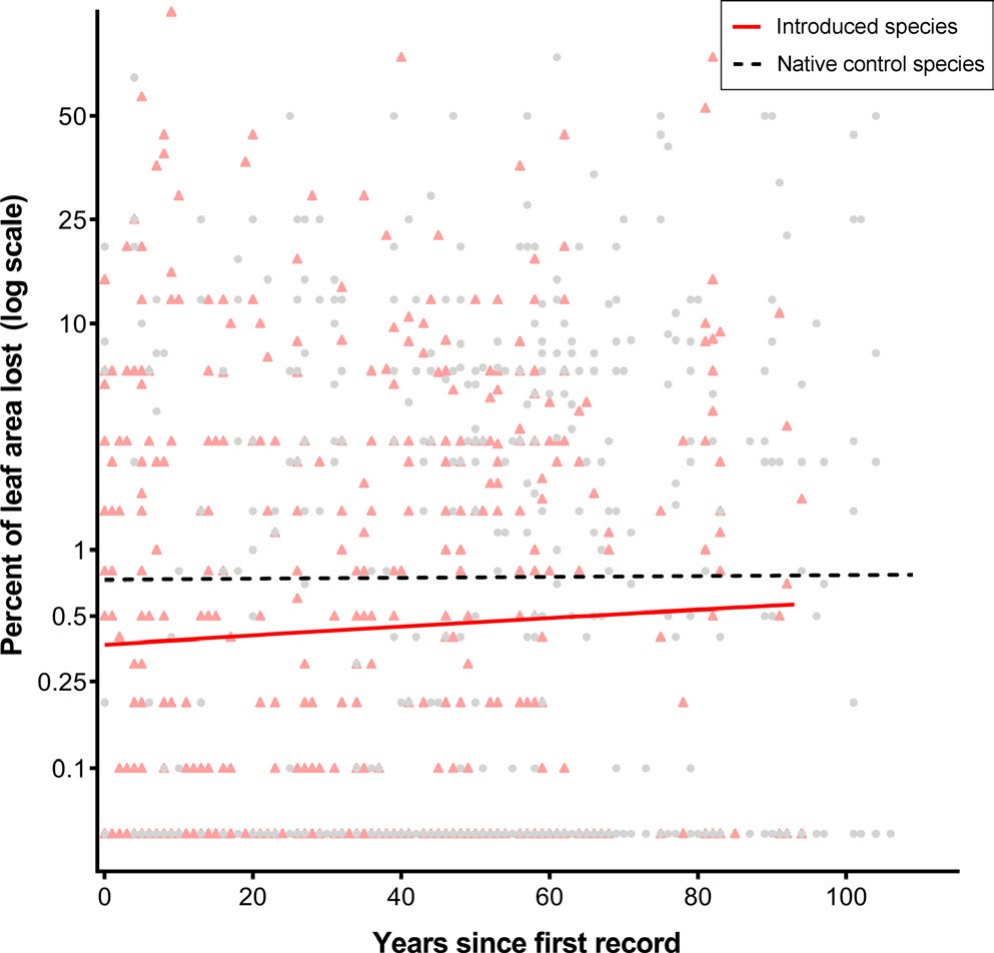
Percentage of leaf area lost (logit transformed) in herbarium specimens of introduced plants (*p* = .20; red triangular data points and red solid line) and native control plants (*p* = .86; gray circular data points and black dashed line) over time

We did not find much evidence for increasing levels of herbivory over time in introduced plants. When we analyzed each species individually, the only introduced species showing a significant change in the level of herbivory experienced with time since arrival was *Parthenium hysterophorus* (*p* = .04, Figure 7). The two‐part hurdle model (Table S4) showed that *P. hysterophorus* had a significantly higher probability of being attacked as time progressed (*p* = .02) but no trend in the amount of leaf eaten following herbivore attack (*p* = .43). An analysis considering the data for all seven introduced species together revealed no significant change in the overall level of herbivory experienced with time since arrival (*p* = .20, Figure 7; Supplementary Data Table S5). Introduced species did not have a higher probability of being attacked as time progressed (*p* = .09) nor did they lose more leaf material to herbivores once they had been attacked (*p* = .55). These findings are not consistent with our hypothesis that herbivory on introduced plant species would increase with time since introduction to a new range. The native control species did not show any change in the levels of herbivory experienced over time either individually (Table S4 and Figure S1) or as a group (*p* = .86, Figure 8). Full model results for all species are available in Tables S4 and S5.

## 4 DISCUSSION

Based on the enemy release hypothesis, we predicted that we would find lower plant defenses and increased herbivory over time in introduced plants. However, despite using the precise and powerful comparison of an introduced plant with its actual source population we found almost no evidence to support these predictions. Surprisingly though, introduced *A. populifolia* had evolved changes in almost every individual defense trait that we measured.

One possible explanation for why we found no overall differences in defense or herbivory in introduced plants could be that changes are occurring more quickly than can be detected by our study. Evidence for rapid evolution in plant and animal populations is steadily accumulating (Hendry & Kinnison, 1999;van Kleunen, Bossdorf, & Dawson, 2018;Reznick & Ghalambor, 2001), with recent studies finding that evolutionary changes in an introduced plant can occur within as little as three years (Sekor & Franks, 2018). Inspection of our data (e.g., *Ageratina*, *Ambrosia* and *Carduus*, Figure 8a,b,e) suggests that some species may experience a short (5–10 year) initial lag phase with little herbivory (neophobia), followed by a sizeable increase in herbivory thereafter. While we found no differences in herbivory between source and introduced plants within the first five years compared to after 50 years (Appendix S1), our data are not particularly well‐suited to addressing this question. Delving further into the question of how defense and herbivory change through the crucial first years after plants are introduced to a new range is an important direction for future work.

Considering how different defense compounds evolve in response to different herbivore types could also help us interpret some of the changes that we found in *A. populifolia*. It has been proposed that introduced plants can undergo evolutionary shifts in the types of defense compounds they produce if they leave their specialist herbivores behind and experience a change to a more generalist herbivore assemblage in their new range (Müller‐Schärer, Schaffner, & Steinger, 2004). Under this scenario, qualitative defenses (e.g., toxins like alkaloids) and quantitative defenses (e.g., digestibility‐reducing defenses like phenolics including tannins) in the home range are expected to be maintained at intermediate levels owing to opposing selection from specialists and generalists, but in the introduced range qualitative defenses are expected to increase and quantitative defenses to decrease in the absence of specialist herbivores (Doorduin & Vrieling, 2011;Müller‐Schärer et al., 2004). Our findings that introduced *A. populifolia* plants have higher levels of alkaloids (a qualitative defense) and lower levels of phenolics (a quantitative defense) lend support to this idea and suggest a possible explanation for some of the chemical changes we observed in the leaves of introduced *A. populifolia*. Additional support for this idea comes from a study on *Senecio jacobaea* (Asteraceae) which found that introduced plants had evolved a 90% increase in pyrrolizidine alkaloids, resulting in increased protection against generalist herbivores but decreased protection against specialist herbivores (Joshi & Vrieling, 2005).

Finally, we consider whether the underlying drivers of some of the trait changes observed in introduced *A. populifolia* may be indicative of other selective pressures. In general, variation in plant traits can result from several different ecological and evolutionary drivers and therefore reflect whole‐plant trade‐offs and strategies (Reich et al., 2003). In the case of *A. populifolia*, introduced and source plants appear to have evolved different defense strategies: Introduced *A. populifolia* plants have relatively tender leaves with higher nitrogen content that are better defended with ash and hairs, as opposed to source *A. populifolia* plants which have comparatively tougher, less‐palatable leaves that are less well‐defended with ash and hairs. High ash content points to a large accumulation of biominerals like calcium oxalates and silica, which are not only used in defense but also play a role in calcium regulation, heavy metal tolerance, and the alleviation of water stress, heat stress, and salinity (Franceschi & Nakata, 2005;Ma, 2004). Increased leaf hair density may have been driven by increased herbivory, but also may be an adaptation to drier or windier conditions in the introduced range (Brandenburger, Cooke, et al., 2019;Ripley, Pammenter, & Smith, 1999). Leaves that become more succulent and less dense may evolve in response to a selection for water‐storing capabilities (Vendramini et al., 2002) but this could in turn lead to leaves that are less tough and therefore more susceptible to herbivory (Hanley et al., 2007). In summary, trait changes that evolve in a new range may be the result of more than just one selective pressure, and this in turn may contribute to some of the inconsistent outcomes obtained in tests of enemy release.

## 5 CONCLUSIONS

Our study has revealed that plants introduced to a new range can adapt extremely rapidly to their new environments. However, these introduced plants may be reaching new defense and herbivory equilibria even before the invader's presence is observed. Exposure to a suite of novel biotic or abiotic factors can result in multiple significant changes in individual defense traits, but no overall change in defense. Some defense trait changes may be driven by environmental factors, but then be offset by other defense trait changes. With so many swings and roundabouts in play, it is not surprising that tests of enemy release have provided such idiosyncratic outcomes in the past. We hope our study might shift the paradigm in invasion ecology away from expecting simple decreases in defense in an introduced range toward acknowledging the fact that defense traits will respond to a whole suite of interacting biotic and abiotic factors in a new range.

## CONFLICT OF INTEREST

None declared.

## AUTHORS’ CONTRIBUTIONS

**Claire R. Brandenburger:** Conceptualization (supporting); Data curation (lead); Formal analysis (lead); Investigation (lead); Methodology (supporting); Project administration (lead); Visualization (lead); Writing‐original draft (lead); Writing‐review & editing (lead). **Martin Kim:** Conceptualization (supporting); Data curation (supporting); Formal analysis (supporting); Investigation (supporting); Methodology (supporting); Project administration (supporting); Writing‐original draft (supporting); Writing‐review & editing (supporting). **Eve Slavich:** Formal analysis (supporting); Methodology (supporting); Software (supporting); Visualization (supporting); Writing‐original draft (supporting); Writing‐review & editing (supporting). **Floret L. Meredith:** Formal analysis (supporting); Funding acquisition (supporting); Investigation (supporting); Methodology (supporting); Resources (supporting); Writing‐original draft (supporting); Writing‐review & editing (supporting). **Juha‐Pekka Salminen:** Formal analysis (supporting); Investigation (supporting); Methodology (supporting); Resources (supporting); Writing‐original draft (supporting); Writing‐review & editing (supporting). **William B. Sherwin:** Conceptualization (supporting); Funding acquisition (supporting); Supervision (supporting); Writing‐original draft (supporting); Writing‐review & editing (supporting). **Angela T. Moles:** Conceptualization (lead); Data curation (supporting); Formal analysis (supporting); Funding acquisition (lead); Investigation (supporting); Methodology (supporting); Project administration (supporting); Resources (supporting); Software (supporting); Supervision (lead); Visualization (supporting); Writing‐original draft (supporting); Writing‐review & editing (supporting).

CRB, ATM, and WS: conception of ideas and methodology design; CRB: plant growth; CRB, MK, FM, and JS: data collection; CRB, ES, ATM, and MK: result analysis; and CRB: writing—manuscript. All authors contributed critically to the drafts.

## Supporting information

Supplementary Material

## Data Availability

Raw data are available for download from the Dryad Digital Repository (https://doi.org/10.5061/dryad.ksn02v716).
