# Supplementary material for: Evolution of defense and herbivory in introduced plants—Testing enemy release using a known source population, herbivore trials, and time since introduction
Source: Ecol Evol. 2020 May 5;10(12):5451–63. doi: 10.1002/ece3.6288 (PMC7319247; doi:10.1002/ece3.6288)
Supplement: Supplementary file 1 — Supplementary Material [file ECE3-10-5451-s001.docx]

**Supporting information**

**Article title:**  Evolution of defence and herbivory in introduced plants – testing enemy release using a known source population, herbivore trials and time since introduction.

**Authors:** Claire R. Brandenburger, Martin Kim, Eve Slavich, Floret L. Meredith, Juha-Pekka Salminen, William B. Sherwin, Angela T. Moles.

The following Supporting Information is available for this article:

**Table S1.** List of introduced study species and native control species with common names, family, introduction dates where applicable and sample sizes for leaf measurements.

**Table S2.** Results of one-way analyses of variance (ANOVAs) contrasting each defence trait among the four introduced populations in Australia.

**Table S3.** Sample sizes for each population and country.

**Table S4.** Model outputs from the two-part hurdle model (probability of damage; amount eaten conditional on non-zero damage) and from the combined model for herbivory over time for individual introduced species and individual native control species.

**Table S5.** Model outputs from the two-part hurdle model (probability of damage; amount eaten conditional on non-zero damage) and from the combined model for herbivory over time for introduced species as a group and native control species as a group.

**Fig. S1**. Percent leaf area lost (logit transformed) in herbarium specimens of three individual native control species in the years since first record.

**Appendix S1.** Testing for a lag phase in herbivory in introduced plants.

**Table S1.** List of introduced study species and native control species with common names, family, introduction dates where applicable (Parsons & Cuthbertson 2001) and the number of leaf measurements taken (sample size).

| **Introduced study species** | **Common name** | **Family** | **Introduction date** | **Sample size** |
| --- | --- | --- | --- | --- |
| *Ageratina adenophora* | Crofton Weed | Asteraceae | 1918 | 265 |
| *Ambrosia psilostachya* | Perennial Ragweed | Asteraceae | 1922 | 215 |
| *Asystasia gangetica* | Chinese Violet | Acanthaceae | 1999 | 60 |
| *Baccharis halimifolia* | Groundsel Bush | Asteraceae | 1941 | 201 |
| *Carduus nutans* | Nodding Thistle | Asteraceae | 1950 | 205 |
| *Gymnocoronis* *spilanthoides* | Senegal Tea Plant | Asteraceae | 1980 | 80 |
| *Parthenium* *hysterophorus* | Parthenium Weed | Asteraceae | 1955 | 135 |
| **Native control species** | **Common name** | **Family** |  |  |
| *Alternanthera* *denticulata* | Lesser Joyweed | Amaranthaceae | NA | 405 |
| *Brunoniella* *australis* | Blue Trumpet | Acanthaceae | NA | 240 |
| *Senecio pinnatifolius* | Coast Groundsel | Asteraceae | NA | 360 |

**Table S2.** Results of one-way analyses of variance (ANOVAs) contrasting each defence trait among the four introduced populations in Australia. Cyanide was not analysed as none of the plants showed cyanogenesis.

| **Trait** | **F** | **p-value** |
| --- | --- | --- |
| Ash | 0.21 | 0.89 |
| Alkaloids | 1.80 | 0.18 |
| C:N ratio | 1.47 | 0.25 |
| Leaf hairs* | 0.43 | 0.73 |
| Phenols | 0.90 | 0.47 |
| Toughness | 1.01 | 0.39 |

*Leaf hair data from (Brandenburger *et al.* 2019)

**Table S3.** Sample sizes (n) for each population and country (SA = the source population in Arniston, South Africa; AUS = Australian totals; M = Mallacoota, AUS; N = Narooma, AUS; T = Treachery Beach, AUS; W = Wairo Beach, AUS).

| **Trait** | **SA (n)** | **AUS (n)** | **M (n)** | **N (n)** | **T (n)** | **W (n)** |
| --- | --- | --- | --- | --- | --- | --- |
| Ash | 14 | 16 | 4 | 4 | 4 | 4 |
| Alkaloids | 22 | 24 | 6 | 6 | 6 | 6 |
| C:N ratio | 24 | 24 | 6 | 6 | 6 | 6 |
| Leaf hairs* | 10 | 34 | 10 | 8 | 7 | 9 |
| Phenols | 20 | 20 | 5 | 5 | 5 | 5 |
| Toughness | 116 | 215 | 69 | 39 | 40 | 67 |

*Leaf hair data from (Brandenburger *et al.* 2019)

**Table S4.** Model outputs from the two-part hurdle model (probability of damage; amount eaten conditional on non-zero damage) and from the combined model for herbivory over time for individual introduced species and individual native control species. Test statistics are log likelihood ratio statistics; odds ratios are the exponentiated coefficient for time in each model. *Only *Parthenium hysterophorus* showed any significant change (p<0.05) in herbivory over time.

|  | **Probability of damage** | | | **Amount eaten conditional on non-zero damage** | | | **Combined model** | |
| --- | --- | --- | --- | --- | --- | --- | --- | --- |
| **Output** | **Test statistic** | **Odds Ratio** | **p-value** | **Test statistic** | **Odds Ratio** | **p-value** | **Test statistic** | **p-value** |
| **Introduced species** | | | | | | | | |
| *A. adenophora* | 0.099 | 1.063 | 0.66 | 0.374 | 1.149 | 0.39 | 0.945 | 0.62 |
| *A. psilostachya* | 0.752 | 1.251 | 0.22 | 0.015 | 0.957 | 0.87 | 1.533 | 0.47 |
| *A. gangetica* | 0.295 | 0.804 | 0.44 | 0.019 | 0.926 | 0.85 | 0.629 | 0.73 |
| *B. halimifolia* | 0.094 | 0.927 | 0.67 | 0.230 | 1.190 | 0.50 | 0.647 | 0.72 |
| *C. nutans* | 0.714 | 1.231 | 0.23 | 0.546 | 1.362 | 0.30 | 2.521 | 0.28 |
| *G. spilanthoides* | 0.024 | 0.951 | 0.83 | 0.563 | 0.725 | 0.29 | 1.173 | 0.56 |
| *P. hysterophorus* | 2.943 | 1.648 | 0.02* | 0.307 | 0.755 | 0.43 | 6.500 | 0.04* |
| **Native control species** | | | | | | | | |
| *A. denticulata* | 0.382 | 0.906 | 0.38 | 0.284 | 0.889 | 0.45 | 1.332 | 0.51 |
| *B. australis* | 0.040 | 1.041 | 0.78 | 0.000 | 0.997 | 0.99 | 0.080 | 0.96 |
| *S. pinnatifolius* | 0.285 | 1.146 | 0.45 | 0.945 | 1.408 | 0.17 | 2.461 | 0.29 |

**Table S5.** Model outputs from the two-part hurdle model (probability of damage; amount eaten conditional on non-zero damage) and from the combined model for herbivory over time for introduced species as a group and native control species as a group. Test statistics are log likelihood ratio statistics; odds ratios are the exponentiated coefficient for time in each model.

|  | **Probability of damage** | | | **Amount eaten conditional on non-zero damage** | | | **Combined model** | |
| --- | --- | --- | --- | --- | --- | --- | --- | --- |
| **Output** | **Odds Ratio** | **Test statistic** | **p-value** | **Odds Ratio** | **Test statistic** | **p-value** | **Test statistic** | **p-value** |
| **Introduced species** | 1.007 | 2.848 | 0.09 | 1.004 | 0.360 | 0.55 | -3.208 | 0.20 |
| **Native control species** | 1.001 | 0.030 | 0.86 | 1.003 | 0.266 | 0.61 | -0.296 | 0.86 |


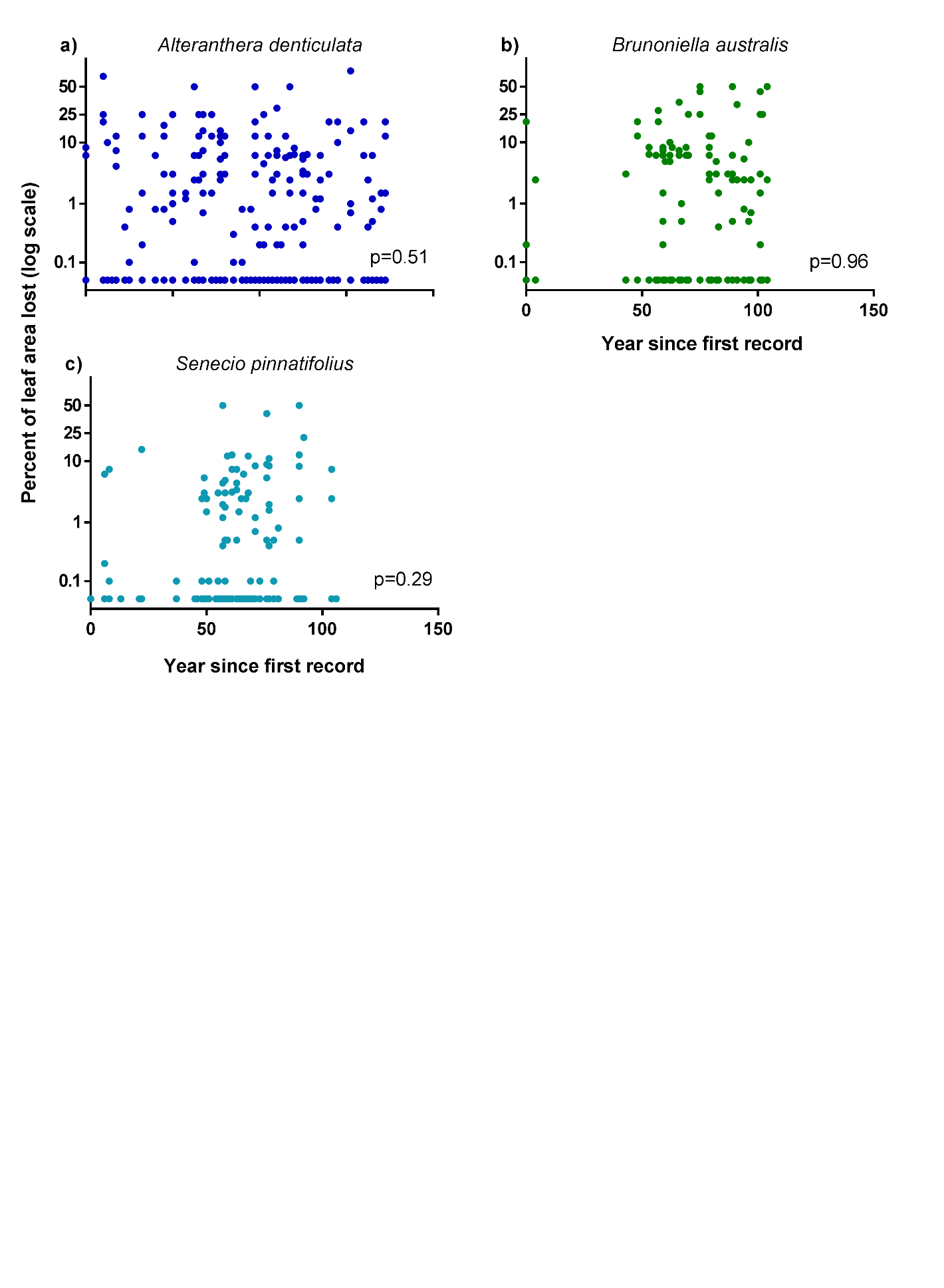


**Fig. S1.** Percent leaf area lost (logit transformed) in herbarium specimens of three individual native control species in the years since first record.

**Appendix S1.** **Testing for a lag phase in herbivory in introduced plants.**

We tested the hypothesis that there are differences in herbivory between introduced species and native species when the introduced species first arrive (within the first five years), but that after time (50 years) there would be no significant difference. Damage was assessed as a proportion of the leaf missing. Many samples contained no damage, and so the prevalence of zeros made it impossible to transform the data to an accessible statistical distribution family. Instead, we created a migration status variable for each sample stating whether it was ‘just arrived <5yrs post introduction’; ‘established migrant >50yrs post introduction’; ‘other’; ‘native’, and then fitted a hurdle model, modelling the probability of leaf damage (binomial response), and the amount of leaf damage conditional on non-zero damage (logit-normal response), with migration status as a fixed effect, and with random effects for species and sheet. The desired contrasts ‘just arrived <5yrs post introduction’ – ‘native’; and ‘established migrant >50yrs post introduction’ – ‘native’ were then tested using the ‘multcomp’ package in R (Hothorn, Bretz & Westfall 2008) which controls the family-wise error rate for each model. None of the comparisons were significant (details provided in Table S6 and S7 below).

**Table S6.** Estimates of the probability of leaf damage from the binary logistic regression- model, with standard errors and p-values.

| **Contrast** | **Estimate (log odds ratio)** | **Standard Error** | **P-value** |
| --- | --- | --- | --- |
| ‘just arrived <5yrs post introduction’ – ‘native’ | 0.260 | 0.331 | 0.64 |
| ‘established migrant >50yrs post introduction’ – ‘native’ | 0.474 | 0.299 | 0.19 |

**Table S7.** Estimates of the amount of leaf damage (logit transformed) conditional on non-zero damage, with standard errors and p-values.

| **Contrast** | **Estimate (logit transform of proportion damaged)** | **Standard Error** | **P-value** |
| --- | --- | --- | --- |
| ‘just arrived <5yrs post introduction’ – ‘native’ | 0.873 | 0.502 | 0.14 |
| ‘established migrant >50yrs post introduction’ – ‘native’ | 0.392 | 0.452 | 0.58 |

# REFERENCES

Brandenburger, C.R., Cooke, J., Sherwin, W.B. & Moles, A.T. (2019) Rapid evolution of leaf physiology in an introduced beach daisy. *Proceedings of the Royal Society B***,** 20191103.

Hothorn, T., Bretz, F. & Westfall, P. (2008) Simultaneous inference in general parametric models. *Biometrical Journal,* **50,** 346-363.

Parsons, W.T. & Cuthbertson, E.G. (2001) *Noxious weeds of Australia,* 2 edn. CSIRO Publishing, Collingwood, Victoria.
